# Smallpox and Vaccination

**Published:** 1921-12

**Authors:** C. Killick Millard

**Affiliations:** (Medical Officer of Health, Leicester)


### SMALLPOX AND VACCINATION.

By C. KILLICK MILLARD, M.D., D.Sc. (Medical Officer of Health, Leicester).

AN important Memorandum on Smallpox and Vaccination has just been issued by the the Ministry of Health,* and circulated to medical officers of health and others.

The actual writer of .the Memorandum is not stated, but, issued as it is under such auspices, it must necessarily be regarded as a weighty and authoritative document. Unfortunately, the subject is such a highly controversial one that it is hardly to be expected—especially in the light of past experience—that anything written on it will be otherwise than *ex parte*, and certainly the present contribution will hardly claim to be impartial. It is frankly a propagandist document, written with the obvious intention of popularising vaccination, and incidentally, no doubt, of furnishing medical officers and others with "authoritative" arguments in favour of infantile vaccination.

For those, however, who believe in the adage *audi alteram partem*, it may not be out of place to make certain observations and criticisms, not in any carping spirit hostile to vaccination, *qua* vaccination, but rather in the belief that the orthodox views about infantile vaccination and its effects upon epidemic smallpox, as expressed in the Memorandum, do not represent quite the whole truth on this complex question.

I venture to suggest, with all respect, that very few medical men are able to approach this subject with unbiassed minds. From the earliest and most susceptible years of their medical career, bias is instilled into them, consciously or unconsciously, by their professional pastors and masters. This may seem a hard thing to say, but it is nevertheless quite true. Moreover, a time-honoured regulation of the General Medical Council—which body has practically absolute power in controlling the medical curriculum—ensures that a final and concentrated dose of bias shall be instilled before any medical student is allowed to qualify. Quite unnecessarily—so far, at least, as learning how to perform the simple operation of vaccination is concerned—every medical student has to take out a special course of instruction in vaccination with a Public Vaccinator, a man who usually receives a substantial part of his professional income from the institution of State Vaccination. All that it is necessary to say about the technique of vaccinating, or about the natural history and pathology of vaccinia, can only fill up a small part of the time set aside for the "course." The rest of the time is largely utilised in extolling the virtues of State Vaccination as an institution, and of showing up the folly of those who doubt them. Is it surprising if much of the instruction given is inspired by the sentiment, "Great is Diana of the Ephesians!"? How infinitely better it would be, so far as the prevention of smallpox is concerned, if the precious time at present devoted by medical students to "learning vaccination" were spent in taking out a special course of instruction in the diagnosis of smallpox. There is no disease in which a mistake in diagnosis may have more disastrous consequences to the community. For it has been proved beyond question that faulty diagnosis has, over and over again, been directly responsible for serious outbreaks of the disease. As Dr. W. McC. Wanklyn says, in his standard work, "The Diagnosis of Smallpox":

> It is essential to recognise the mischief which may be done by unrecognised cases of smallpox. There is hardly any disease of which the prompt recognition is more important to the general community. Almost every outbreak in London in recent years has been started, or propagated or prolonged, by unrecognised cases. Epidemics teem with examples which only cease to be recorded because they become trite.

The Memorandum begins with a reference to the liability of this country to the introduction of smallpox from abroad, and to the fact that epidemics may vary greatly in type. A brief description is given of smallpox in pre-vaccination times, and we are told how universal the disease was in the eighteenth century. Reference is made to the altered age-incidence of the disease, and we are told that in pre-vaccination days the mortality fell chiefly upon the early years of life. This, of course, is only what we should expect to be the case with a disease from which almost everyone suffered and which conferred practically lifelong immunity. Older persons were either naturally immune or protected by an attack early in life. It is not necessary to assume, as we are sometimes told, that smallpox is essentially a disease of childhood. On the contrary, there are good reasons for thinking that under modern conditions (even apart from vaccination) smallpox is rather a disease of adults. In Leicester there has been no special incidence upon children. Thus, out of over 700 cases of smallpox occurring in the years 1903-21, only 155 children were attacked, of whom 10 died.

Then follows a brief account of smallpox inoculation, and of the effects of the disease in causing disfigurement. The amazing statement is made, without any qualification, that

> in pre-vaccination days men declined to marry maidens who had not had smallpox. The maid who had passed through an attack of smallpox possessed many advantages not enjoyed by her sister who had not had the disease. She was not likely to have a second attack, and her chance of living to a reasonable age could be estimated with some degree of certainty. True, she might not have escaped entirely unscathed, for few ever did who recovered; her beauty might be impaired, her skin deeply pitted and scarred, and her features partly destroyed. These conditions were preferable, however, to blindness, deafness, and permanent invalidism, which too often resulted from an attack of smallpox.

* "Reports on Public Health and Medical Subjects. No. 8, Smallpox and Vaccination." Price 3d. H.M. Stationery Office.

**Smallpox and Vaccination—(*cont.*).**

This passage has been quoted *in extenso* because it is a good example of the blood-curdling sort of stuff which the present writer was taught as a medical student when "learning vaccination," and which he faithfully believed until he was forced to re-interpret it in the light of actual experience of smallpox in a practically unvaccinated city (Leicester). He now knows that sometimes smallpox is very severe, but often it is mild, even in the unvaccinated. To suggest that in pre-vaccination days smallpox was nearly always of a severe type, and usually produced the horrible effects so luridly depicted above, is certainly an exaggeration and unworthy of a scientific document. Of course, it is easy to understand that the more serious and fatal epidemics were the ones that made the deepest impressions, and so have come down to us in the pages of literature. This is what we generally find. It is the exceptional and arresting facts that get recorded, whilst the usual and commonplace are quickly forgotten.

We are next given a brief description of vaccinia and of the discovery and history of vaccination. To prevent any possible misapprehension the present writer wishes to say at once that he appreciates fully the epoch-making character of this discovery, and regards it as quite the most brilliant in the whole history of preventive medicine. Would that other "vaccines" had an equally potent effect in immunising the individual against other diseases!

On p. 5 the influence of improved sanitation is discussed, and I am pleased to note that more credit is allowed to this than was at one time usual. It is admitted that "overcrowding, uncleanliness of persons and their surroundings favour the spread of infection"; that our water supplies are purer and our sewerage systems better; that Public Health Acts have been passed and health officers appointed. As a set-off against the argument that improved "sanitation"* can largely account for the reduction of smallpox, we are reminded that measles and whooping-cough have been little affected. A few years ago any suggestion that the decline in smallpox mortality could be due to any influence other than infantile vaccination was scouted by the orthodox as rank heresy. To-day, however, faced as we are with the quite unexpected phenomenon that the decline in smallpox has continued without interruption or set-back in spite of the fact that infant vaccination is being more and more neglected, it is obviously necessary to reconsider orthodox doctrine on this point.

As regards the value of the hospital isolation of smallpox, whilst this is admitted, we are warned that smallpox hospitals have often proved a source of danger to the neighbourhood, and that sanitary authorities have not, and cannot provide, sufficient accommodation for any possible emergency "in a population entirely unprotected by vaccination." Of course, the remedy for danger of spread of infection arising from a smallpox hospital is to have the hospital situated at a sufficient distance from centres of population; whilst as for the second point, it raises the whole question whether, under modern conditions of smallpox prevention, there is really more danger of a widespread epidemic of smallpox in an entirely unvaccinated community than in communities composed chiefly of partially devaccinated persons, which is the condition of all communities where infant vaccination (not followed by revaccination) prevails.

Even in well-vaccinated districts widespread and fatal epidemics of smallpox may occur and hospital accommodation prove insufficient. This has happened repeatedly in the past. In such a contingency exceptional measures will need to be taken with a view to providing emergency hospital accommodation. In the case of small districts, or, indeed, of any district in danger of being overpowered, the Ministry of Health should promptly give assistance, for an uncontrolled outbreak of smallpox is a national danger which threatens the whole country. The offer of portable hospitals, ready complete with all equipment and the necessary staff, would be of infinite value.

It would be a comparatively simple matter for the Ministry of Health to make provision for such portable hospitals as I have in mind for use in emergency in whatever part of the country they may be required. If they are never required, then all the better. The cost would be trifling compared with the national expenditure on infantile vaccination.

Before leaving this point, let me observe that infant vaccination, no matter how thoroughly it be carried out, will never obviate the necessity for hospital accommodation when an outbreak occurs, any more than it will prevent outbreaks occurring. In Middlesbrough, in 1897, a fatal and widespread epidemic occurred (1,411 cases), in spite of the fact that the Vaccination Acts had been carried out exceptionally thoroughly. The medical officer of health estimated the proportion of unvaccinated persons in the population to be only about 2 per cent. Yet in that epidemic no less than 108 vaccinated persons lost their lives! The reason why Middlesbrough suffered so severely was certainly not neglect of infantile vaccination. The real reasons were (1) the insanitary condition of the town, (2) insufficient hospital accommodation.

On p. 11 reference is made to the Vaccination Act of 1898, which first recognised and gave exemption to the "conscientious objector." We are told that Lord Lister, in opposing the Bill in the House of Lords, described it as a "tremendous experiment." As a matter of fact, medical authorities throughout the country were almost unanimous in their opposition, and prophesied that the result of the experiment would be disastrous. They said that it would lead to a great diminution in the amount of vaccination, and, as a necessary consequence, to a great increase in the mortality from smallpox. The first part of the prophecy has come quite true. There has undoubtedly been a very great falling-off in vaccination, a large and steadily increasing propor-

* I use the term in its broadest sense to include notification, isolation, supervision of contacts, etc.

**Smallpox and Vaccination—(*cont.*).**

tion of parents taking advantage of the provisions of the Act to avoid having their children vaccinated. But, contrary to all medical expectations, the increase in the mortality from smallpox has not taken place. On the other hand, smallpox has continued to decline *pari passu* with the decrease in vaccination, and within the past ten years it has become practically negligible as a cause of death. Such a totally unexpected result should surely give us pause and make us inquire whether the relationship between the prevalence of smallpox and the absence of infant vaccination is quite so close as has generally been supposed.

On page 12 we are told that when a smallpox epidemic does occur there is a rush for vaccination, and that such "panic" vaccination is apt to be followed by "bad arms" and untoward results. We are not told, however, that the rush to get vaccinated, when even a trifling epidemic occurs, is generally the result of the policy pursued by the authorities of deliberately endeavouring to work up a smallpox scare, and so to frighten people into getting vaccinated. It frequently happens that the epidemic of vaccinia, thus artificially produced, is really much more serious, so far as its actual effects go, than the outbreak of smallpox; e.g., there may be a few dozen cases of mild smallpox, with three or four deaths (in the case of the City of Nottingham recently there was not a single death), whilst the epidemic of vaccinia may run into tens of thousands.

Of course, if it be really true that the epidemic of vaccinia prevented the small outbreak of smallpox from becoming a very large outbreak, it would be quite justified, but there are reasons for doubting this. Vaccination to be effective needs to be applied "at the spot." Thus vaccination of all who come in contact with the sufferers, such as health officials, nurses, and also contacts, is very effective. The experience of Leicester, almost an unvaccinated town so far as general vaccination of population is concerned, seems to indicate that such restricted vaccination is sufficient. When vaccination is used in this way you get the maximum result with the minimum amount of vaccination, and therefore with the minimum amount of inconvenience and injury to health.

In conclusion, no reference is made in the Memorandum to the remarkable and very significant fact that during recent years, owing to the great decrease in smallpox, there have actually been fewer deaths in children under five years from smallpox than from vaccination! To give the actual figures, during the thirteen years 1908-1920, there have been in the whole of England and Wales twenty-five deaths from smallpox in children under five, whilst there have been 111 deaths caused by or associated with vaccination! It is possible, of course, that some few of the latter should not have been attributed to vaccinia, but at least they were officially certified as such by qualified medical practitioners.

No reference is made in the Memorandum to one serious drawback to infantile vaccination which is now beginning to be recognised, viz., its effect in "masking" smallpox, and thus rendering its early recognition very much more difficult. Very many outbreaks have owed their origin to this. Space does not permit of my elaborating this aspect of the question, but certainly it ought to be taken into the reckoning when balancing the advantages and disadvantages of our system of State vaccination. The anti-vaccinists have never been able to make use of this argument against vaccination, because they will not admit that vaccination has the effect of modifying smallpox. Thereby they have deprived themselves of what might have been one of their most potent arguments in fighting compulsory infantile vaccination.

It is beyond question that our methods of combating smallpox in this country in recent years have been greatly perfected, and consequently the danger of epidemic smallpox has been enormously reduced. No one who has studied the subject can doubt this. I believe that the danger has been so far reduced that the time has come when the question of the necessity for infantile vaccination should be reconsidered. Personally I believe it is no longer true to say either (1) that unvaccinated children run a very serious danger of contracting smallpox, or (2) that unvaccinated persons are a serious danger to other people. Yet it was upon these two grounds that compulsory infantile vaccination was originally justified.

---

### A CHRISTMAS CARNIVAL.

All kinds of attractions are promised at the Christmas Carnival and Children's Fair, to be held at the Royal Albert Hall from December 26 to January 4, in aid of, and organised by, the following societies: Cheyne Hospital for Children; Church of England Waifs and Strays; City of London Maternity Hospital; East London Hospital for Children; Hospital for Sick Children, Great Ormond Street; Invalid Children's Aid Association; London Lock Hospital (Infants' and Children's Section); National Institute for the Blind; "Sunshine" Homes for Blind Children; Royal Waterloo Hospital for Women and Children; "Save the Children" Fund (British Section); and Victoria Hospital for Children. That an entertainment which is to benefit suffering childhood should take the form of an ideal Christmas treat for children is a charming idea.

Some of the delights in store at the Albert Hall for our young friends are a "Lilliputian circus"; an "all-star" variety show and juvenile play; a dance of live toys; children's sports, organised by Miss Ellaline Terriss; and a giant Christmas-tree. The price of admission is but 1s. 3d. Dancing, on the celebrated floor used at the famous balls, will take place each afternoon and evening (tickets, 2s. 6d.). The Fair will be open daily from 1.30 to 10 p.m., with two special events—a great children's party on December 29 from 2.30 to 6.30 (5s.), and a Cinderella dance on New Year's Eve from 7 to 12 p.m. (5s.).

Tickets may be obtained from Mrs. C. F. Sargon, Office of East London Hospital for Children, 199 Piccadilly, W. 1; from Mrs. Claremont, M.B.E., National Institute for the Blind, 224-8 Great Portland Street, W. 1; and from the secretaries of the above societies.

